# Supplementary material for: Delta-dependent Notch activation closes the early neuroblast temporal program to promote lineage progression and neurogenesis termination in Drosophila
Source: eLife. 2024 Feb 23;12:RP88565. doi: 10.7554/eLife.88565 (PMC10942576; doi:10.7554/eLife.88565)
Supplement: Supplementary file 1. — Table of genotypes listed by panel of each figure and figure supplement. [file elife-88565-supp1.docx]

**Supplemental Table 1: *Drosophila* genotypes per figure panel**

| **Figure** | **Genotype** | | | | |
| --- | --- | --- | --- | --- | --- |
| Figure 1B | *worGAL4/+ (OregonR) ; pcnaGFP/+ (OregonR)* | | | | control |
| Figure 1C | *worGAL4/+; pcnaGFP/UAS-NotchRNAi* | | | | |
| Figure 1F | *hsflp, tubgal80, FRT19A/Notch55e11 FRT19A ; tubGal4, UAS-mCD8GFP/+* | | | | |
| Figure 1G,I | *worGAL4/+; pcnaGFP/UAS-KuzRNAi* | | | | |
| Figure 1H | *worGAL4/+; pcnaGFP/UAS-Su(H)RNAi* | | | | |
| Figure 2A,B,C | *NP0577GAL4/+; UAS-mCD8RFP/+; DeltaGFP/+* | | | | |
| Figure 2D,E,F | *repoGAL4, UAS-mCD8RFP/SerrateGFP* | | | | |
| Figure 2G | *worGAL4/+; E(spl)mγGFP/+* | | control | | |
| Figure 2H | *worGAL4/+; E(spl)mγGFP/UAS-NotchRNAi* | | | | |
| Figure 2I | *worGAL4/+; E(spl)mγGFP/UAS-DeltaRNAi* | | | | |
| Figure 2J | *repoGAL4/+; E(spl)mγGFP/+* | | control | | |
| Figure 2K | *repoGAL4/+; E(spl)mγGFP/UAS-DeltaRNAi* | | | | |
| Figure 2L | *repoGAL4/+; E(spl)mγGFP/UAS-SerrateRNAi* | | | | |
| Figure 3A | *worGAL4/+; pcnaGFP/UAS-DeltaRNAi* | | | | |
| Figure 3B | *worGAL4/+; pcnaGFP/UAS-DeltaRNAi* | | | | |
| Figure 3E | *NP0577GAL4/+; pcnaGFP/UAS-DeltaRNAi* | | | | |
| Figure 4A | *worGAL4,tubGAL80(ts)/+; pcnaGFP/UAS-NotchRNAi* (left panel) | | | | |
| Figure 4A | *worGAL4,tubGAL80(ts)/+; pcnaGFP/UAS-DeltaRNAi* (right panel) | | | | |
| Figure 4B | *worGAL4,tubGAL80(ts)/+; pcnaGFP/UAS-NotchRNAi* (left panel) | | | | |
| Figure 4B | *worGAL4,tubGAL80(ts)/+; pcnaGFP/UAS-DeltaRNAi* (right panel) | | | | |
| Figure 5C | *worGAL4/+ (OregonR)* | | control | | |
| Figure 5D | *worGAL4/+; UAS-NotchRNAi/+* | | | | |
| Figure 5E | *worGAL4/+; UAS-DeltaRNAi/+* | | | | |
| Figure 5F | *ImpGFP/+ (OregonR); worGAL4/+ (OregonR)* | | control | | |
| Figure 5G | *ImpGFP/+; worGAL4/+; UAS-NotchRNAi/+* | | | | |
| Figure 5H | *ImpGFP/+; worGAL4/+; UAS-DeltaRNAi/+* | | | | |
| Figure 5J | *hsFlp; repoGAL80; UAS-DeltaRNAi/Act5c-FRT-CD2-FRT-Gal4, UAS-RFP* | | | | |
| Figure 6A | *hsflp, tubgal80, FRT19A/Notch55e11 FRT19A ; tubGal4, UAS-mCD8GFP/+* | | | | |
| Figure 6B | *worGAL4/+; UAS-DeltaRNAi/+* | | | | |
| Figure 6C | *worGAL4/+; pcnaGFP/UAS-ImpRNAi* | | | | |
| Figure 6D | *pcnaGFP; worGAL4,tubGAL80(ts)/+; UAS-DeltaRNAi/UAS-ImpRNAi* | | | | |
| Figure 6E | *worGAL4/+; pcnaGFP/UAS-Eip93F WT* | | | | |
| Figure 6F | *pcnaGFP; worGAL4,tubGAL80(ts)/+; UAS-DeltaRNAi/UAS-ImpRNAi* | | | | |
| Figure 7D | *DeltaGFP/+* | control | | | |
| Figure 7E | *worGAL4/+; DeltaGFP/UAS-ImpRNAi* | | | | |
| Figure 7F | *worGAL4/UAS-Syp-RB-HA; DeltaGFP/+* | | | | |
| Figure 7G | *worGAL4/+; DeltaGFP/UAS-Eip93F WT* | | | | |
|  | | | | | |
| **Supplementary Figures** | | | | | |
| Figure 4-figure supplement 1A,B | *worGAL4,tubGAL80(ts)/+; E(spl)mγGFP/UAS-DeltaRNAi* | | | | |
| Figure 5-figure supplement 1A | *ImpGFP/+ (OregonR); worGAL4/+ (OregonR)* | | | control | |
| Figure 5-figure supplement 1B | *ImpGFP/+; worGAL4/+; UAS-NotchRNAi/+* | | | | |
| Figure 5-figure supplement 1C | *ImpGFP/+; worGAL4/+; UAS-DeltaRNAi/+* | | | | |
| Figure 5-figure supplement 1E | control: *worGAL/+ (Oregon R)*  *worGAL4/+; UAS-DeltaRNAi/+* | | | | |
| Figure 5-figure supplement 1F | *prosGAL4/+; UAS-DeltaRNAi/+* | | | | |
| Figure 5-figure supplement 1G | *worGAL4,UAS-dp110/+; UAS-DeltaRNAi/+* | | | | |
| Figure 6-figure supplement 1A | *worGAL4/+; pcnaGFP/UAS-ImpRNAi* | | | | |
